# P-292. Epidemiology of *Escherichia coli* from Urine Sources at Three U.S. Sites, August 2023

**DOI:** 10.1093/ofid/ofae631.495

**Published:** 2025-01-29

**Authors:** Joshua M Brandenburg, Julian E Grass, Hsioa Che Looi, Julia Tellerman, Jenna Dietz, Paulina Rebolledo, Gillian Smith, Jesse T Jacob, Erin Parker, Rebecca Longson, Alice Guh, Heather N Grome

**Affiliations:** Centers for Disease Control and Prevention, Atlanta, Georgia; CDC, Atlanta, GA; New York Rochester Emerging Infections Program at the University of Rochester Medical Center, Rochester, New York, Rochester, New York; New York Emerging Infections Program, Rochester, New York; Center for Community Health and Prevention, Rochester, New York; Emory University School of Medicine, Emory University Rollins School of Public Health, Atlanta, GA; Georgia Emerging Infections Program, Atlanta, GA; Foundation for Atlanta Veterans Education and Research, Decatur, GA; Atlanta Veterans Affairs Medical Center, Decatur, GA, Atlanta, Georgia; Emory University School of Medicine, Atlanta, GA; California Emerging Infections Program, Oakland, California; California Emerging Infections Program, Oakland, California; Centers for Disease Control and Prevention, Atlanta, Georgia; Centers for Disease Control and Prevention, Atlanta, Georgia

## Abstract

**Background:**

*Escherichia coli* is the most frequent cause of urinary tract infections (UTIs) in the US, but surveillance for urinary *E. coli* (uEC) is often limited to multidrug-resistant strains. The CDC’s Emerging Infections Program piloted one month of active population- and laboratory-based surveillance for uEC of any phenotype in 3 U.S. sites (representing >3.4 million people) to better characterize its epidemiology and burden.

**Methods:**

We defined an incident uEC case as the first *E. coli* isolated from urine in a resident of the surveillance area during August 2023. Demographic data were obtained for all incident cases. Approximately 400 cases per site were randomly selected for chart review. One-month incidence rates were generated using 2022 US census data for participating surveillance areas. Cases were classified as community-associated (CA; no healthcare risk factors); hospital-onset (HO; urine obtained ≥ 3 days after hospital admission), or healthcare-associated, community onset (HACO; urine obtained in a non-hospital setting or < 3 days after hospital admission with healthcare exposures in the prior year).

**Results:**

Of 5241 uEC patients, 86.1% were female; median age was 60 years (IQR 37–76). The 1-month incidence rate was 151.7 per 100,000 population and greater among those aged ≥ 60 vs < 60 years (362.7 vs 94.0).

Of 1197 patients with data from chart review, 1104 (92.2%) had cultures collected as an outpatient; 732 (61.2%) had an underlying comorbidity reported; 1113 (93.0%) had a documented UTI diagnosis; 269 (22.5%) reported recurrent UTI; and 43 (3.6%) developed *E. coli* bacteremia. No signs or symptoms associated with uEC were reported for 372 (31.1%) patients. In total, 287 (24%) were hospitalized within 30 days after urine culture collection; 51/287 (17.8%) had a urinary catheter in place 2 days before culture collection. Most uEC were CA (867; 72.4%), followed by HACO (325; 27.2%) and HO (3; 0.3%).

Of 3289 uEC isolates with local testing results, 255 (7.8%) were extended-spectrum β-lactamase producing and 5 (< 1%) were carbapenem-resistant.

**Conclusion:**

These findings emphasize the high burden of uEC in these communities, notably among older adults and females. Effective prevention strategies are needed to decrease uEC-associated morbidity.

**Disclosures:**

**All Authors**: No reported disclosures

